# Physiomorphic and molecular-based evaluation of wheat germplasm under drought and heat stress

**DOI:** 10.3389/fpls.2023.1107945

**Published:** 2023-04-12

**Authors:** Hameed Alsamadany, Yahya Alzahrani, Zahid Hussain Shah

**Affiliations:** ^1^ Department of Biological Sciences, Faculty of Science, King Abdulaziz University, Jeddah, Saudi Arabia; ^2^ Department of Plant Breeding and Genetics, Pir Mehar Ali Shah Arid Agriculture University, Rawalpindi, Pakistan

**Keywords:** biochemical contents, physiological parameters, plant–water relations, growth and yield traits, gene expression

## Abstract

Drought and heat stress are potential problems that can reduce wheat yield, particularly during the terminal growth stages in arid and semiarid regions of the world. The current study intended to examine the impact of individual and combined drought and heat stress on the biochemical contents (antioxidant enzymes, proline, soluble proteins, and soluble sugars), physiological parameters (chlorophyll content, cell membrane stability, photosynthesis, stomatal conductance, and transpiration), plant–water relations (relative water content, water potential, osmotic potential, and pressure potential), agronomic traits (flag leaf area, plant height, number of tillers per plant, spike length, grains per spike, and thousand-grain weight), and gene expression (*TaHSF1a, TaWRKY-33, TaNAC2L*, and *TaGASR1*) in four different thermostable and drought-tolerant wheat genotypes (i.e., Gold-16, HS-240, Suntop, and Hemai-13) collected from different countries. The tri-replicate experiment was conducted using two factorial arrangements in a randomized complete block design (RCBD). All measured traits, except total soluble sugars, proline, and cell membrane stability index, showed significant reduction under both combined and individual treatments. Furthermore, correlation analysis revealed a significant association between biochemical and physiological characteristics and crop agronomic productivity. Furthermore, principal component analysis (PCA) and heatmap analysis demonstrated significant levels of variation in traits according to the type of stress and nature of wheat genotype. The spectrographs and micrographs generated by scanning electron microscopy for the selected high- and low- tolerance samples revealed clear differences in mineral distribution and starch granulation. All studied genes showed comparatively high levels of relative expression under combined treatments of drought and heat stress in all wheat genotypes, but this expression was the highest in ‘Gold-16’ followed by ‘HS-240’, ‘Suntop’, and ‘Hemai-13’. Overall, this study concluded that plants are proactive entities and they respond to stresses at all levels; however, the tolerant plants tend to retain the integrity of their biochemical, physiological, and molecular equilibrium.

## 1 Introduction

Wheat is an important cereal crop, cultivated worldwide on 200 million hectares, and is the third most cultivated crop after rice and maize (Coffel et al., 2018). Every year abiotic stresses, such as drought, heat, and salinity, cause significant losses in wheat production (Lamaoui et al., 2018; Lan et al., 2022). Climatic and environmental problems significantly reduce wheat production (Asseng et al., 2011). For example, drought stress impairs wheat growth at all stages of its phenological development; however, the extent of vulnerability varies from stage to stage (Djanaguiraman et al., 2020). In arid and semiarid regions, high temperatures and droughts cause a significant reduction in wheat yield at the grain-filling stage (Shah et al., 2022). The main effects of high temperatures include the disruption of plant–water relations and physiological processes, such as photosynthesis, stomatal conductance, chlorophyll concentration, and membrane integrity, causing a significant decline in productivity (Sehgal et al., 2018). Furthermore, high temperatures lead to a shorter life cycle, pollen abortion, anther indehiscence, a reduced grain number, and a reduced grain weight, ultimately causing an overall decrease in agronomic yield (Sehgal et al., 2018; Djanaguiraman et al., 2020). In addition, both drought and heat stress imbalance osmolyte concentration, disturb physiological processes, and affect morphological and yield parameters such as flag leaf area (FLA), spike length, and grain weight (Chaudhary et al., 2020; Pandey et al., 2022). Moreover, drought and heat stress decrease chlorophyll content and overall photosynthetic productivity, increase leaf senescence, promote carbohydrate mobilization to the stem, and reduce the grain-filling duration, resulting in stem weight loss in addition to a decline in grain number per spike, grain weight, straw yield, and harvest index (Sarto et al., 2017; Alghabari et al., 2021).

The harmful effects of drought on wheat are exacerbated further when it occurs simultaneously with heat stress (Coffel et al., 2018). Together with heat stress, drought stress drastically affects various plant processes with high intensity, which can vary according to crop, genotype, and growth stage (Sattar et al., 2020). Owing to global warming and hazardous climate change patterns, it is essential that the impacts of heat stress, and possible procedures for enhancing wheat production under such extreme conditions, are investigated (Asseng et al., 2011). In the context of possible climate change, various environmental stresses have garnered great interest owing to their diverse impacts on crop production; therefore, various models for the adaptation of crops could be adopted by understanding the dynamics of climate change impacts (Fahad et al., 2017). Some other adaptive strategies, which plants can adopt when growing under drought and heat extremes, include the alteration of osmoprotectant and antioxidant contents (Djanaguiraman et al., 2018; Djanaguiraman et al., 2020). Furthermore, abiotic stresses trigger the production of reactive oxygen species (ROS); therefore, understanding the role of antioxidant enzymes, such as catalases (CATs), superoxide dismutases (SODs), and peroxidases (PODs), is very important in this context (Djanaguiraman et al., 2018). The reduced activities of antioxidant enzymes during drought and heat stress triggers the production of ROS, which in turn disrupts membrane fluidity and integrity, and, consequently, causes further electrolyte leakage (Shah et al., 2017).

On the other hand, owing to differences in genetic makeup, plants respond in different ways to drought and heat stress; hence an understanding of gene expression is very important during stress conditions for identifying resistant or susceptible genotypes (Ni et al., 2018). For instance, the over-expression of heat shock factors (*Hsf*) plays a vital role in providing heat and drought stress tolerance in wheat, owing to their role as an osmoprotectant (Bi et al., 2020). In addition, the expression pattern of the wheat *TaGASR1* gene strongly modulates under drought, heat, and oxidative stress, probably owing to its involvement in the induction of the abscisic acid (ABA)-mediated signaling pathway that triggers the stress response process in plants (Zhang et al., 2017). Similarly, genes from the *WRKY* family are induced under heat and drought stress owing to their tendency to activate an antioxidant response, as confirmed in model plants such as *Arabidopsis* and rice (Niu et al., 2012). In addition, plant NAC (no apical meristem) (i.e., NAM, ATAF1/2, and CUC2) proteins play an important role in determining plant responses to biotic and abiotic stresses, for example over-expression of the *TaNAC2L* gene in wheat and transgenic *Arabidopsis* under heat and drought stress indicates its role in the modulation of the expression of other stress-associated genes (Guo et al., 2015).

Drought and heat stress are potential threats in arid and semiarid regions of the world that drastically impact wheat productivity, particularly at the anthesis and grain-filling stages; therefore, sustainable agriculture necessitates the introduction of thermostable wheat germplasm in these affected areas (Alghabari et al., 2021). The present study intended to evaluate the performance of wheat genotypes collected from different institutions around the world under the individual and combined application of drought and heat stress at physiomorphic and molecular levels to determine their potential use for future cultivation in the arid conditions of Saudi Arabia.

## 2 Materials and methods

In the current study, four thermostable and drought-resistant wheat cultivars, ‘Gold-16’ (Pakistan), ‘Suntop’ (Australia), ‘Hemai-13’ (China), and ‘HS-240’ (India), were evaluated using a two-factorial arrangement in a randomized complete block design (RCBD) under control, heat, drought, and combined heat and drought treatments at the experimental area of the Department of Biological Sciences, King Abdulaziz University, Jeddah, Saudi Arabia. The tri-replicate pot experiment was conducted in a single run within a greenhouse.

### 2.1 Crop husbandry and treatment application

Crop husbandry practices were conducted following the procedure used by Schmidt et al. (2020). The pot experiment was conducted in a greenhouse where plants were grown in pots with a substrate mix containing coco peat, clay loam, and sand in a 1 : 1 : 1 ratio with basal slow-releasing nitrogen, phosphorus, and potassium fertilizer. Eight surface-sterilized seeds were sown in each 3-L pot and five pots were used for each treatment. Weeding and hoeing were performed throughout the growing season. Thinning was practiced when plants reached seedling stage and five plants were kept in each pot. Pots were kept under well-watered conditions until the plants attained the stage of anthesis. Plants were provided with isolated and combined drought and heat treatments 3 days after the anthesis of the primary tiller. Drought was achieved by stopping irrigation for 6 days, while heat stress was implemented on the fourth day of drought application by exposing plants to day and night temperatures of 35°C and 25°C, respectively, in heat chambers with 16-hour light (300 μmol m^–2^ s^–1^) and 8-hour dark periods, and a constant 70% humidity. Data for biochemical, physiological, and molecular traits were collected from five randomly selected plants from each treatment every 4 days from the application of stress until plants reached physiological maturity at the reproductive stage. The data for growth and yield parameters were taken after crop harvesting. The impact of positional error was minimized by repositioning the pots on an alternate basis.

### 2.2 Evaluation of biochemical traits

The activities of antioxidant enzymes, such as superoxide dismutase (SOD), peroxidase (POD), and catalase (CAT), were measured using the method described by Djanaguiraman et al. (2020). For the purpose of the study, 2 g of homogeneous frozen leaf samples were mixed in 2 mL of ice-cold 0.1 M Tris-HCl buffer. Subsequently, centrifugation was performed at 4°C and 2,000 for 15 minutes. Afterward, the supernatant was collected, and enzymatic activity was estimated using an SOD assay kit (Sigma-Aldrich, USA), POD assay kit (BiolabsInc, USA), and CAT assay kit (Sigma-Aldrich, USA), following the instructions of the manufacturers. Total soluble sugars (TSSs) were determined by using the method described by Shi et al. (2016). For this purpose, leaf samples were ground to a fine powder and mixed with 80% ethanol. Afterward, the mixture was centrifuged at 5,000 g for 10 minutes. This method was conducted three times and each time the supernatant was collected. The resulting pooled sample was further diluted with 80% ethanol and TSSs were determined using a standard curve, with absorbance being measured at 625 nm in the presence of the anthrone reagent. Proline content was measured following the protocol used by Ábrahám et al. (2010) and quantified using a UV–Vis spectrophotometer (Edinburgh Instruments, UK) based on its rate of reaction with ninhydrin. The concentration of total soluble protein (TSP) was measured using the Bradford Assay (Bradford, 1976) and quantification was carried out with the help of a UV–Vis spectrophotometer (Edinburgh Instruments, UK).

### 2.3 Estimation of physiological parameters

Chlorophyll content was estimated following the method adopted by Sattar et al. (2020). Transpiration (Tr), stomatal conductance (Gs), and photosynthesis (Pn) were estimated using Syrus 3 (Decagon Devices, model SC-1, 2011). In addition, cell membrane stability (CMS) was calculated according to the method described by ElBasyoni et al. (2017), using the formula:


(1) $$CMS=\{[1-(X_{1}/X_{2})]/[1-(Y_{1}/Y_{2})]\}\times 100,$$

where X_1_ is the conductance of an un-autoclaved and stress-exposed plant sample; X_2_ is the conductance of an autoclaved and stress-exposed plant sample; Y_1_ is the conductance an un-autoclaved of control plant sample; and Y_2_ is the conductance of an autoclaved control plant sample.

### 2.4 Estimation of plant–water relations

The relative water content (RWC) of the leaves was measured following the procedure adopted by Shah et al. (2022), using the formula:


(2) $$RWC=[(W_{Fresh}-W_{Dry})/(W_{Total}-W_{Dry})\times 100].$$

Plant–water relations, such as water potential (ψ_w_), osmotic potential (ψ_s_), and pressure potential (ψ_p_), were estimated following the method adopted by Sattar et al. (2020). The water potential of leaves (ψ_w_) was measured using a Scholander bomb (PMS Instrument Company, Albany, USA), following the instructions given. Osmotic potential (ψ_s_) was calculated with the help of an osmometer (Advanced Instruments, Norwood, USA), following the set procedure. Leaf pressure potential (ψ_p_) was determined as the difference between ψ_w_ and ψ_s._

### 2.5 Measurement of growth and yield traits

For the measurement of growth attributes, flag leaf area (FLA) was recorded as per the scale used by Farooq et al. (2009), while the average plant height (PH) of randomly selected plants was measured from the shoot to the apex at maturity using a ruler. The number of tillers per plant (NTP) was calculated as an average from randomly selected plants of all genotypes. For the yield traits, mean spike length (SL) was calculated with the help of a numeric scale from randomly selected plants. Afterward, the number of grains per spike (GPS), for all genotypes, was estimated. Thousand-grain weight (TGW) was recorded using an electronic weighing balance (Bioevopeak, Jinan, China).

### 2.6 Scanning electron microscopy

The scanning electron microscope (SEM) spectrograph for elemental distribution and micrograph for starch granulation were generated by placing selected samples in the Energy Dispersive X-ray (EDX) system of the scanning electron microscope, which was equipped with autoquantification and autoidentification features. In the spectrograph, the peaks’ height quantified the elements, whereas the peaks’ position identified the elements.

### 2.7 Analysis of gene expression

For expression analysis of heat- and drought*-*related gene expression (*TaHSF1a*, *TaWRKY-33*, *TaNAC2L*, and *TaGASR1*), an RNA extraction kit (Sigma-Aldrich, USA) was used to extract total RNA from selected leaf samples of wheat genotypes following the standard procedure adopted by Li et al. (2019). Subsequently a complementary DNA (cDNA) library was prepared according to the method used by Ahmed et al. (2022). For this purpose, 2 μg of total RNA was used and quantitative real-time PCR (qRT-PCR) analysis was carried out. In addition, the TaActin1-expressing gene was used to normalize the relative expression of the aforementioned genes. The sequences of the primers used in gene expression analysis are presented in **Table 1**.

**Table 1:** Primers used in the estimation of relative gene expression.

Gene	Primer (5′–3′)
*TaHSFA1a*	AGCATTCCAGGATTCCCAGAT (F) CCAGGCATTGCGAAATTCTC (R)
*TaWRKY-33*	GGCTTCAACGGCAACTTCG (F) ATGTCCTCCTCCCTCGGCTC (R)
*TaNAC2L*	CAGAGACAGAGATCGACAGAAG (F) GAGCTACATCCGCATTGAGAG (R)
*TaGASR1*	GACTTCATCCCCATCCTCCG (F) CGTTGTCCTCGTTGATCTCCC (R)

F, forward; R, reverse.

### 2.8 Statistical analysis

The data collected after the implementation of treatments were analyzed using computer-based statistics programs. The software Statistix8.1 was used for ANOVA at a *p*-value ≤ 0.05, while the packages of R, version 4.1.0, (The R Foundation for Statistical Computing, Vienna, Austria) were used for principal component analysis (PCA), correlation analysis, and heatmap analysis.

## 3 Results

### 3.1 Biochemical contents

**Figure 1:**
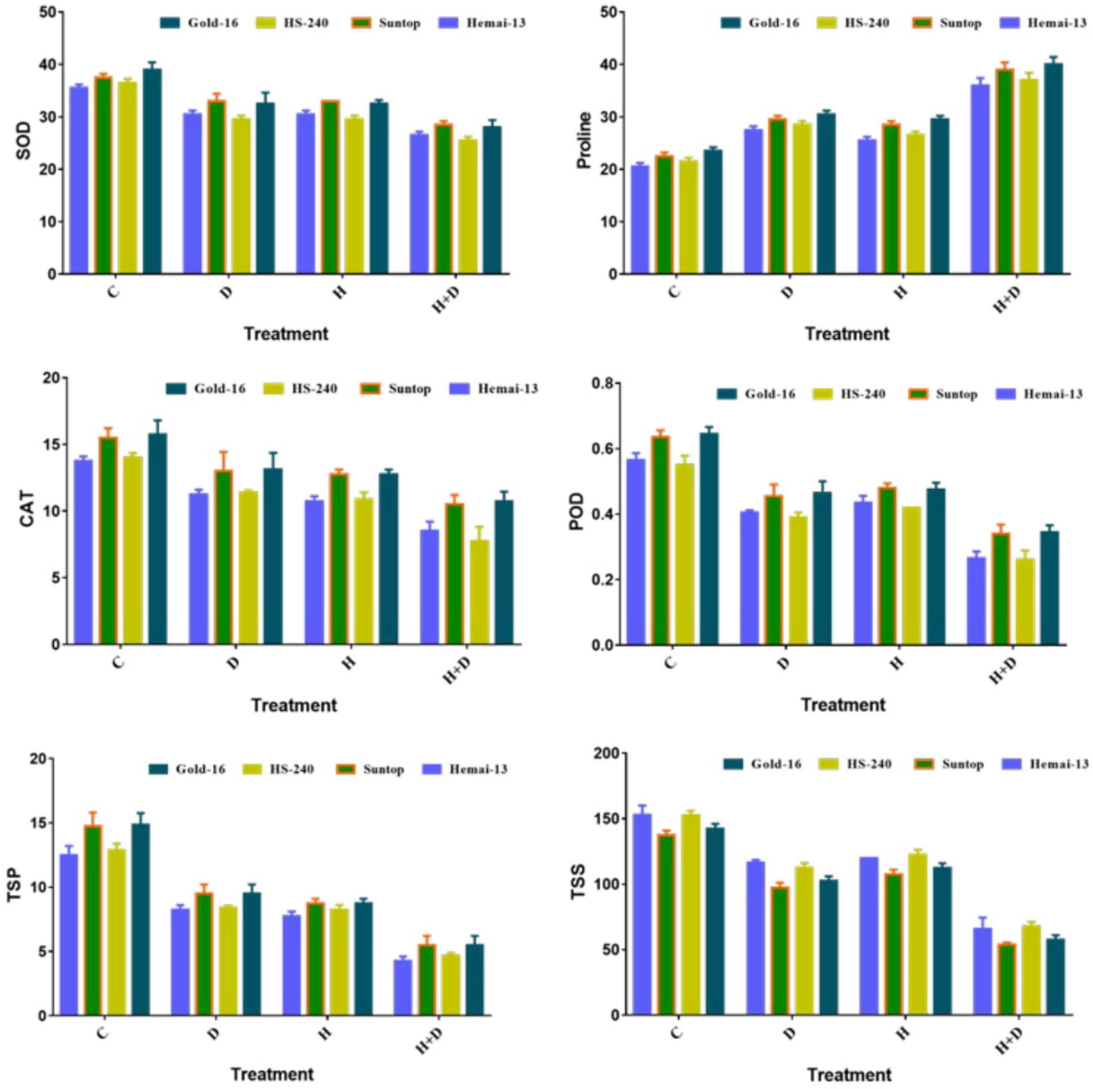
Variation in biochemical contents of wheat cultivars due to individual and combined treatments of drought and heat stress. Dry weight (DW); fresh weight (FW); superoxide dismutase (SOD); catalase (CAT); peroxidase (POD); total soluble proteins (TSPs); total soluble sugars (TSSs). Values in the graph are means averaged after every 4 days of treatment application during the two-factorial tri-replicate experiment at a *p*-value ≤ 0.05. *Units: POD, CAT, and CAT activities (enzyme units); proline (μgg^–1^FW); TSP (mgg^–1^DW); TSS (mgg^–1^DW).

Both separated and combined treatments of drought and heat stress significantly (*p *≤ 0.05) impacted all biochemical traits, including SOD, POD, CAT, TSSs, TSP, and proline. All genotypes recorded a significant decrease in the activities of SOD, POD, and CAT, and concentration of TSP under both individual and combined treatments of heat and drought stress (**Figure 1**). This reduction was more dramatic when the plants were grown under the combined application than the individual applications of drought and heat stress. In addition, isolated treatments of drought and heat stress demonstrated analogous effects. Among the different genotypes, Hemai-13 illustrated the highest, whereas Gold-16 illustrated the lowest, reduction in the activities of antioxidant enzymes and TSP under both individual and combined treatments of heat and drought stress (**Figure 1**). Contrary to the aforementioned biochemical traits, proline and TSSs recorded statistically distinct increases in all genotypes during both individual and combined applications of drought and heat stress, compared with the control treatment (**Figure 1**). Correspondingly, this increase was more dramatic in the combined treatment and was similar between the individual applications of drought and heat stress (**Figure 1**). Among the different genotypes, Gold-16 recorded the highest, whereas Heami-13 recorded the lowest, increase in proline content. Conversely, Hemai-13 demonstrated the greatest, whereas Gold-16 demonstrated the smallest, increase in TSS content (**Figure 1**).

### 3.2 Physiological parameters

**Figure 2:**
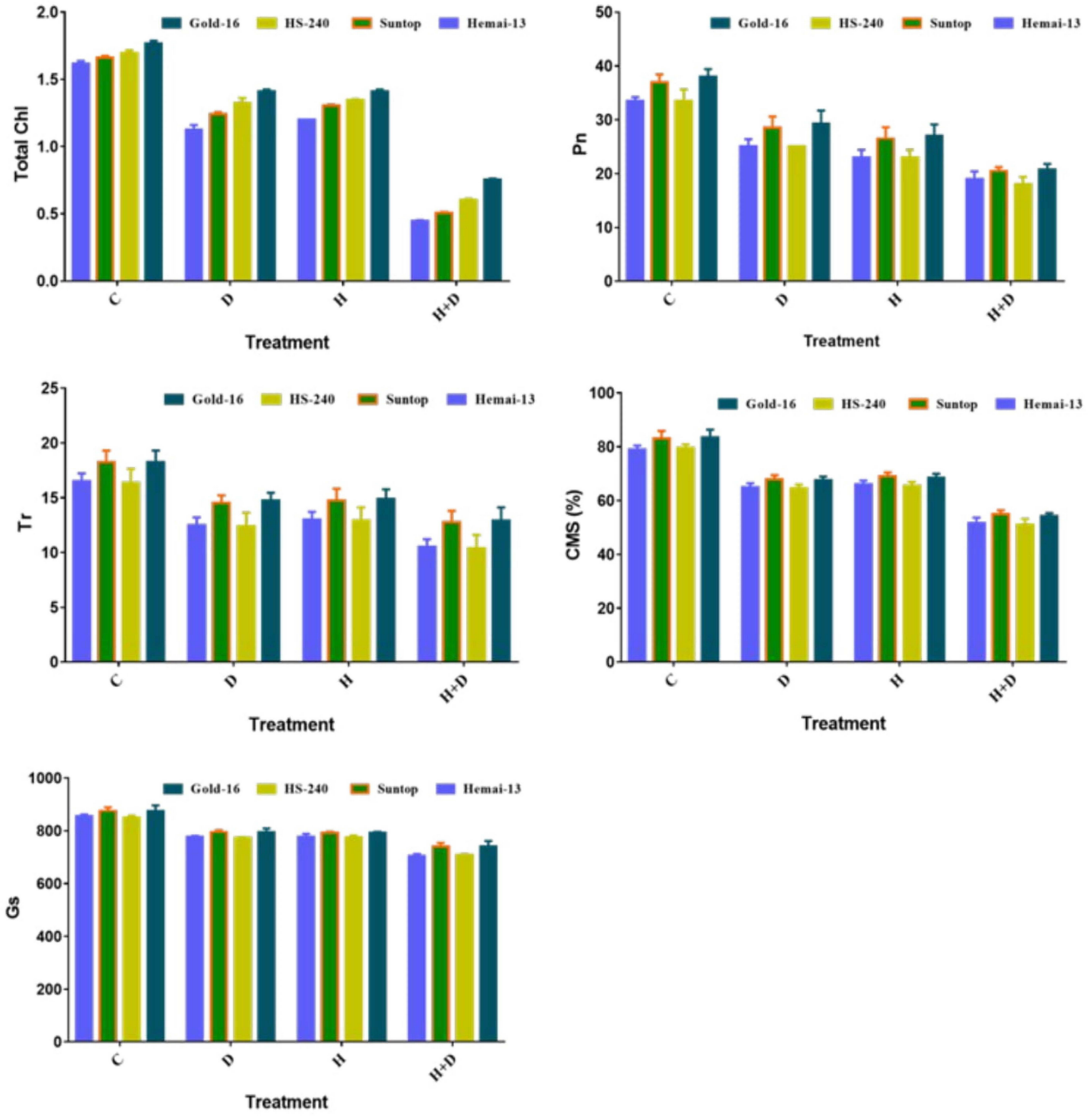
Variation in physiological attributes of wheat cultivars due to individual and combined treatments of drought and heat stress. Photosynthesis rate (Pn); transpiration (Tr); cell membrane stability (CMS); stomatal conductance (Gs). Values in graph are means averaged after every 4 days of treatment application during the two-factorial tri-replicate experiment at a *p*-value ≤ 0.05. *Units: Total chlorophyl l content (g kg^–1^); Pn (μm^–2^S^–1^); Tr (mmm^–2^S^–1^); Gs (mmm^–2^S^–1^).

All treatments had a significant (*p* ≤ 0.05) effect on the physiological traits, including chlorophyll content, photosynthesis rate (Pn), transpiration rate (Tr), cell membrane stability (CMS), and stomatal conductance (Gs), in all genotypes compared with the control treatment (**Figure 2**). All genotypes showed a statistically distinct reduction in physiological traits under both single and combined treatments of drought and heat stress; however, this reduction was far greater under the combined application of drought and heat stress than under the individual applications (**Figure 2**). In addition, both drought and heat stress led to a corresponding decline in the mean values of all these physiological attributes. Among the different genotypes, Gold-16 recorded the lowest decrease in chlorophyll content, Pn, Tr, CMS, and Gs, followed by HS-240, then Suntop, and then Hemai-13 (**Figure 2**).

### 3.3 Plant–water relations

**Figure 3:**
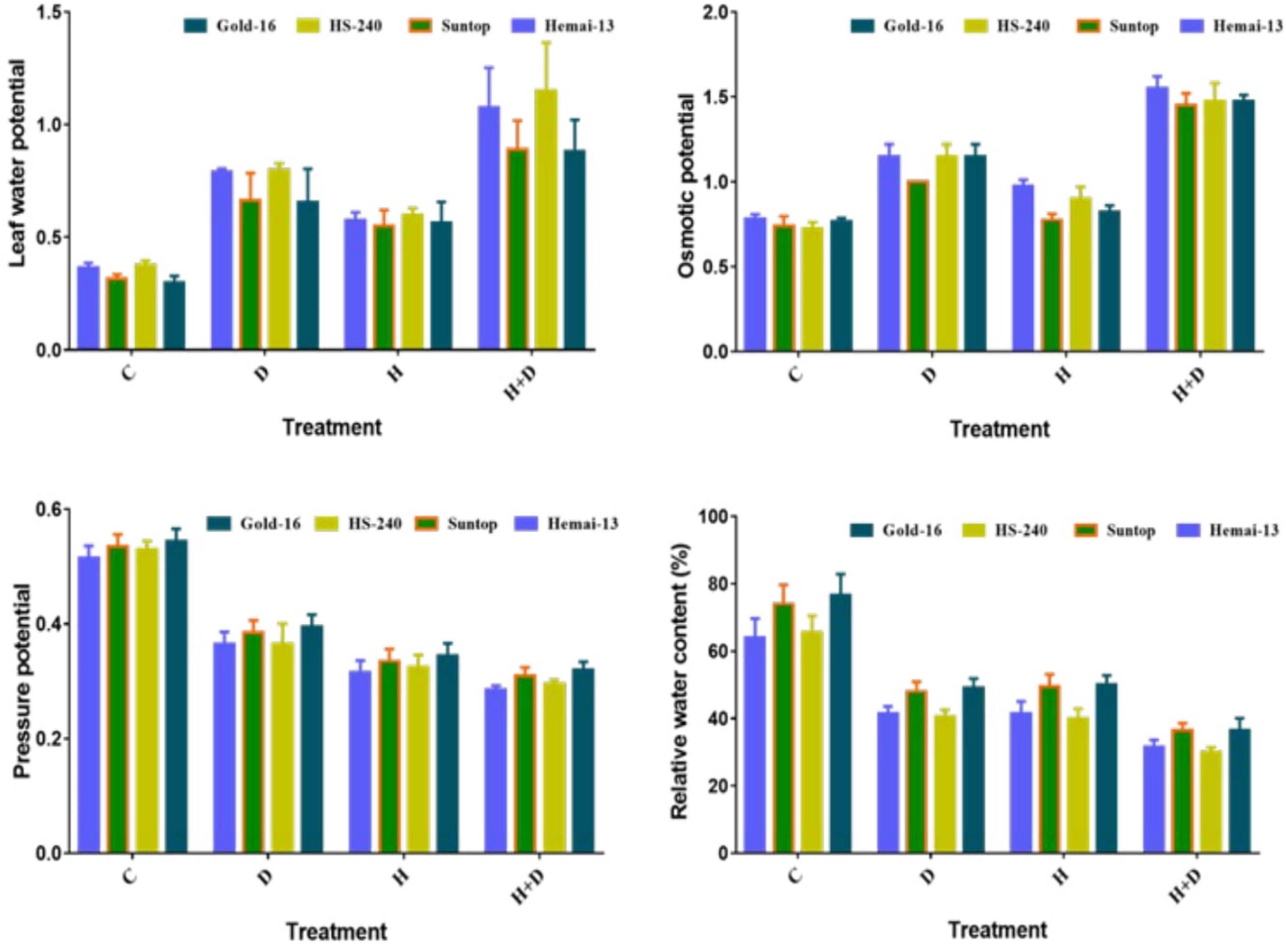
Variation in plant–water relations of wheat genotypes due to individual and combined treatments of drought and heat stress. Leaf water potential (ψ_w_); osmotic potential (ψ_s_); pressure potential (ψ_p_); relative water content (RWC). Values in the graph are means averaged after every 4 days of treatment application during the two-factorial tri-replicate experiment at a *p*-value ≤ 0.05. *Units: ψ_w_ *(–MPa);* ψ_s_ *(MPa);* ψ_p_ *(MPa)*.

All water-related parameters, such as RWC, water potential (ψ_w_), osmotic potential (ψ_s_), and pressure potential (ψ_p_), showed significant (*p *≤ 0.05) variations associated with both individual and combined regimes of drought and heat stress in all wheat genotypes (**Figure 3**). The RWC, water potential, and pressure potential demonstrated a significant decrease under both isolated and combined regimes of drought and heat treatments compared with the control treatment; however, this decrease was more dramatic in the combined treatment (**Figure 3**). Among the different genotypes, Hemai-13 showed the greatest decrease in the aforementioned traits, followed by Suntop, then HS-240, and then Gold-16. In addition, osmotic potential demonstrated a statistically distinct increase in all genotypes in both the individual and combined regimes of drought and heat stress compared with the control treatment (**Figure 3**). Correspondingly, this increase was more dramatic under the combined application of treatments. Among the different genotypes, Hemai-13 recorded the greatest rise while Gold-16 recorded the lowest rise in osmolytes, as revealed by their respective osmotic potential values (**Figure 3**).

### 3.4 Growth and yield traits

**Figure 4:**
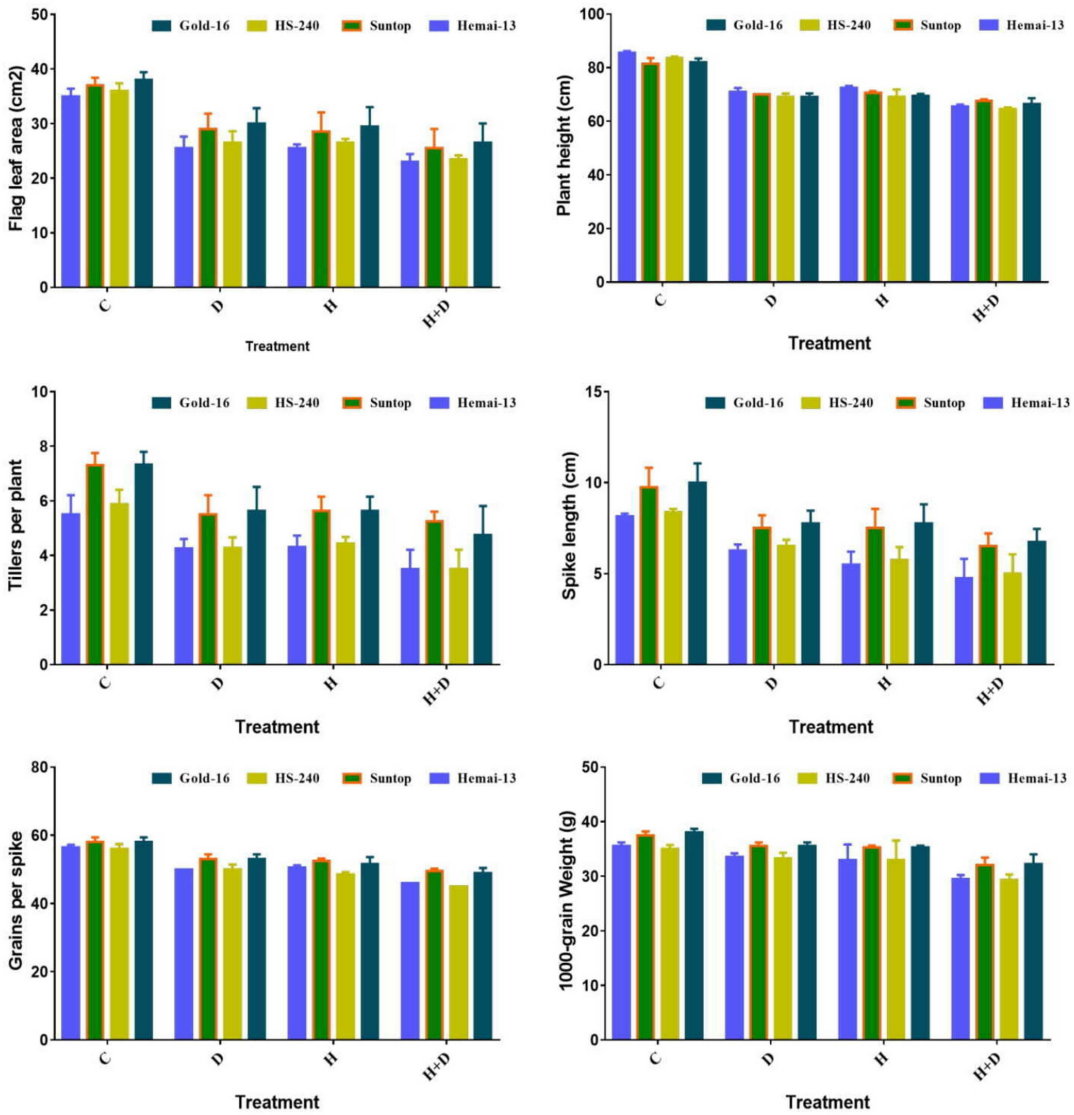
Variation in growth and yield traits of wheat cultivars due to individual and combined treatments of drought and heat stresses. Values in the graph are means averaged after harvesting during the two-factorial tri-replicate experiment at a *p*-value ≤ 0.05.

All treatments significantly (*p *≤ 0.05) altered all agronomic traits, including flag leaf area (FLA), plant height (PH), number of tillers per plant (NTP), spike length (SL), grains per spike (GPS), and thousand-grain weight (TGW). All these traits demonstrated a statistically significant reduction under both separate and combined treatments of drought and heat stress; however, this reduction was more dramatic for the combined treatment of stresses (**Figure 4**). Among the different genotypes, Hemai-13 showed the greatest reduction in all aforementioned growth and yield traits, followed by Suntop, then HS-240, and then Gold-16, under both individual and combined regimes of drought and heat stress (**Figure 4**).

### 3.5 Correlation analysis, PCA, and heatmap analysis

**Figure 5:**
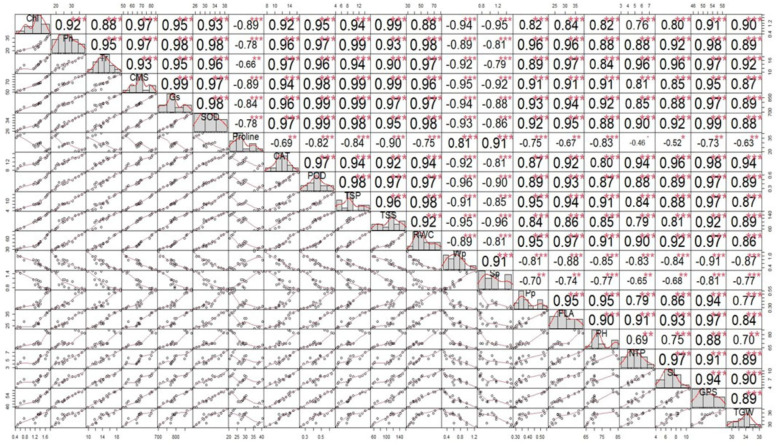
Correlation matrix showing the extent of association between the biochemical contents, physiological attributes, plant–water relations, and agronomic traits in wheat cultivars and individual and combined treatments of heat and drought stress. Superoxide dismutase (SOD); catalase (CAT); peroxidase (POD); total soluble proteins (TSPs); total soluble sugars (TSSs), photosynthesis rate (Pn); transpiration rate (Tr); cell membrane stability (CMS), stomatal conductance (Gs); water potential (Wp); osmotic potential (Sp); pressure potential (Pp); relative water content (RWC); flag leaf area (FLA); plant height (PH); number of tillers per plant (NTP); spike length (SL); number of grains per spike (GPS); and thousand-grain weight (TGW). ***= Significant at P ≤ 0.001; **= Significant at P ≤ 0.01; *= Significant at P ≤ 0.05.

The correlation analysis revealed a significant paired association among plant–water relations, biochemical contents, physiological attributes, and agronomic traits; however, the extent of the association was different in the single and combined drought and heat stress treatments than in the control treatment (**Figure 5**). Among the physiological traits, chlorophyll content demonstrated a highly significant positive paired association with CMS, PH, SL, ψ_w_, and TGW under the combined application of drought and heat stress compared with the individual stress and control treatments (**Figure 5**). Moreover, FLA showed a more significant positive correlation with Pn, Gs, Tr, ψ_w_, and ψ_p_ under the combined stress treatment than the control treatment. Similarly, RWC demonstrated a significantly higher correlation with CMS, Tr, ψ_w_, and ψ_p_ under both single and combined stress treatments than the control treatment (**Figure 5**). Similarly, the activity of antioxidant enzymes SOD, POD, and CAT showed a significant positive association with all traits except ψ_s_, TSSs, and protein under both the combined and individual application of treatments, compared with the control treatment (**Figure 5**). Conversely, TSSs, proline, and ψ_s_ demonstrated a significantly negative paired association with other traits and a significantly positive correlation between each other under the combined and individual application of stresses, when compared with the control treatments (**Figure 5**).

**Figure 6:**
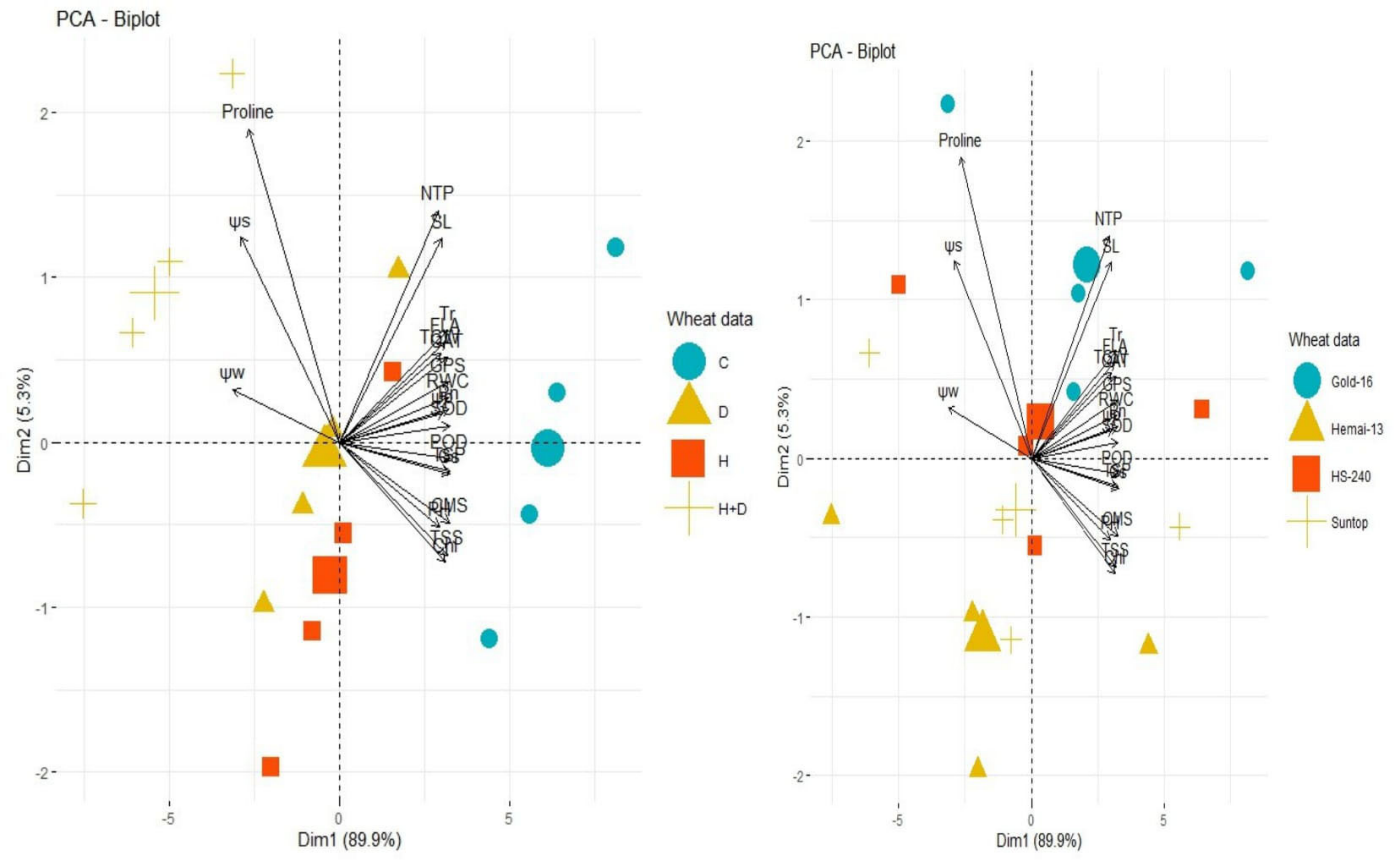
Principal component analysis (PCA) vectors demonstrating the effect on the proximity association between biochemical contents, physiological attributes, plant–water relations, and agronomic traits, and wheat cultivars (right) and individual and combined treatments of heat and drought stress (left). Superoxide dismutase (SOD); catalase (CAT); peroxidase (POD); total soluble proteins (TSPs); total soluble sugars (TSSs), photosynthesis rate (Pn); transpiration rate(Tr); cell membrane stability (CMS); stomatal conductance (Gs); water potential (Wp); osmotic potential (Sp); pressure potential (Pp); relative water content (RWC); flag leaf area (FLA); plant height (PH); number of tillers per plant (NTP); spike length (SL); number of grains per spike (GPS); and thousand-grain weight (TGW).

**Figure 7:**
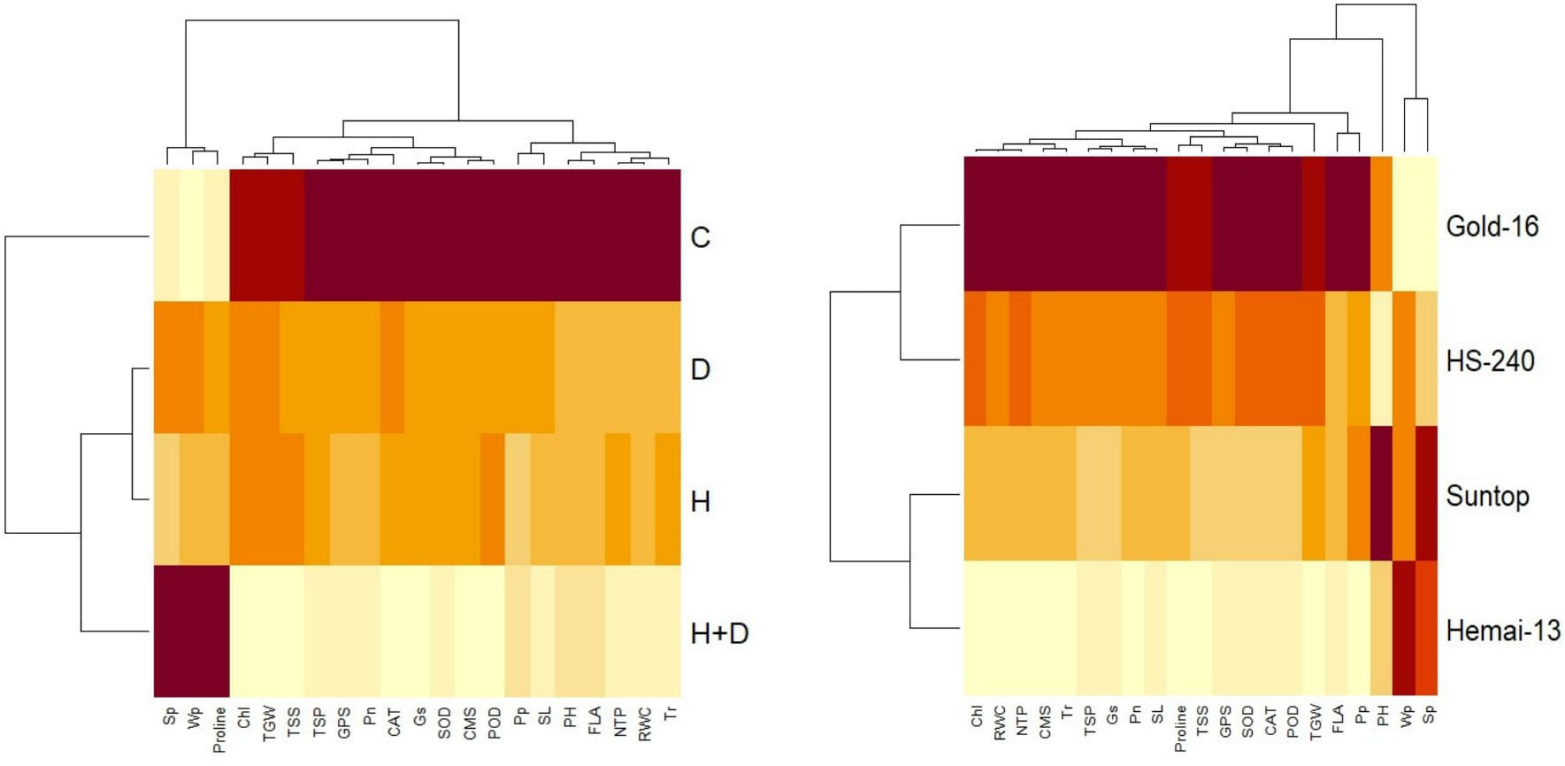
Heatmap cluster analysis depicting varying expression and association patterns of biochemical contents, physiological attributes, plant–water relations, and agronomic traits of wheat cultivars (right) and individual and combined treatments of heat and drought stress (left). Superoxide dismutase (SOD); catalase (CAT); peroxidase (POD); total soluble proteins (TSPs); total soluble sugars (TSSs); photosynthesis rate (Pn); transpiration rate (Tr); cell membrane stability (CMS); stomatal conductance (Gs); water potential (Wp); osmotic potential (Sp); pressure potential (Pp); relative water content (RWC); flag leaf area (FLA); plant height (PH); number of tillers per plant (NTP); spike length (SL); number of grains per spike (GPS); and thousand-grain weight (TGW).

In contrast, PCA revealed the differential dispersion of traits with respect to origin under individual, combined, and treatments (**Figure 6**). Furthermore, variation in orientation of traits in clusters of the PCA graph with respect to treatments indicated change in expression and association pattern of all traits. All genotypes revealed comparative differences in the orientation of trait clusters in the PCA graph under individual and combined applications of drought and heat stress that demonstrate a different stress response tendency in each genotype (**Figure 6**). Heatmap analysis further validated these findings by categorizing traits into different clusters according to the difference in the extent of their association with respect to genotypes and response under the individual and combined application of drought and heat stress (**Figure 7**).

### 3.6 Scanning electron microscopy

**Figure 8:**
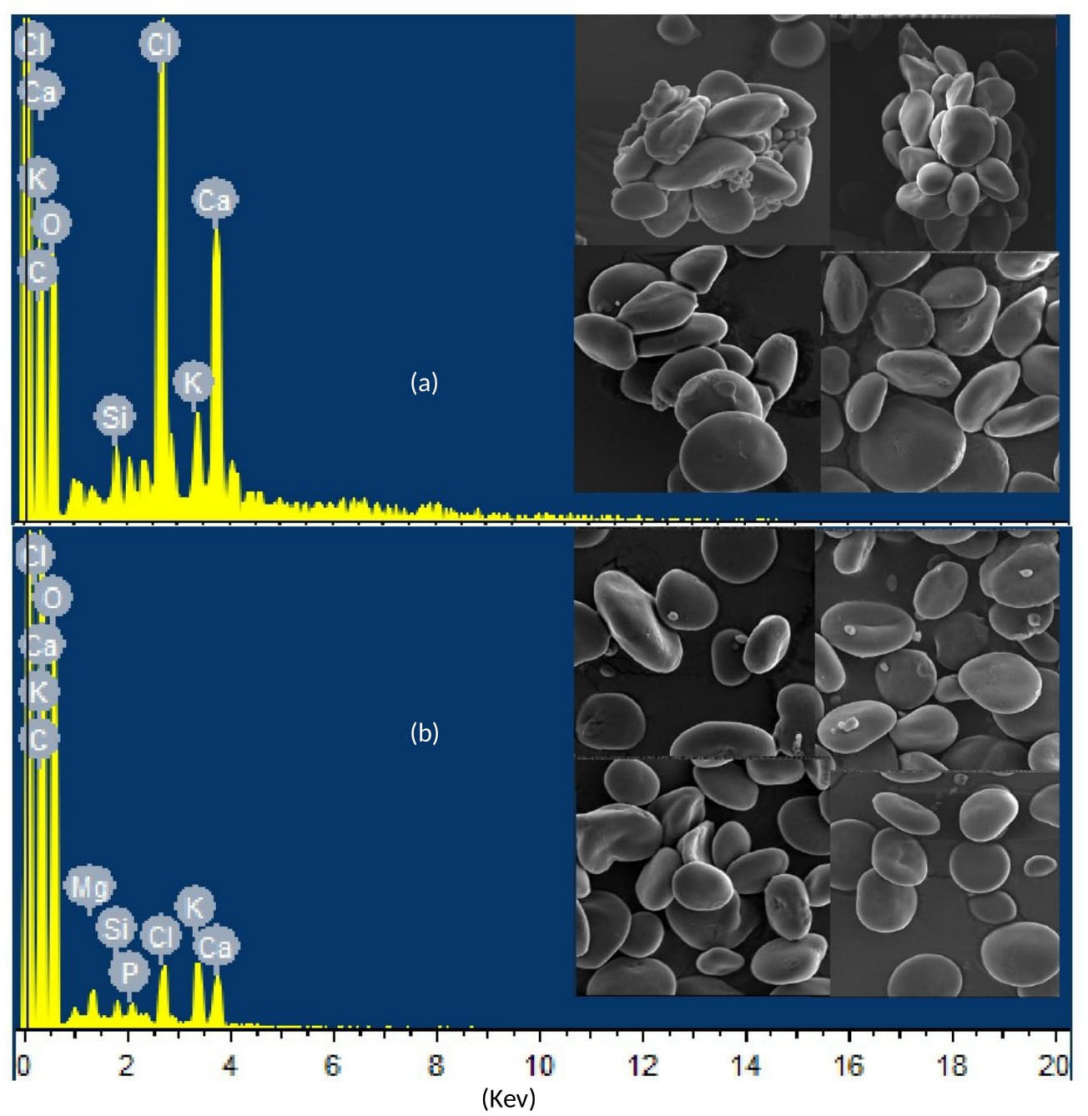
Scanning electron microscope (SEM) spectrographs and micrographs showing differential elemental distribution and starch granulation, respectively, in representative samples of wheat genotypes Gold-16 and Hemai-13, respectively, under **(A)** the control treatment and **(B)** combined drought and heat stress.

The SEM micrographs of selected samples revealed a clear difference in elemental distribution and starch granulations in individual and combined treatments of drought and heat stress compared with the control treatment. The differences in the height and position of peaks in the SEM spectrograph indicate differences in quantity and type of elements, respectively, assimilated in leaves during individual and combined applications of drought and heat treatments. In addition, the integrity of starch granules was highly disrupted in leaves subjected to combined drought and heat stress when compared with those receiving the control treatment, as shown in **Figure 8**.

### 3.7 Gene expression analysis

**Figure 9:**
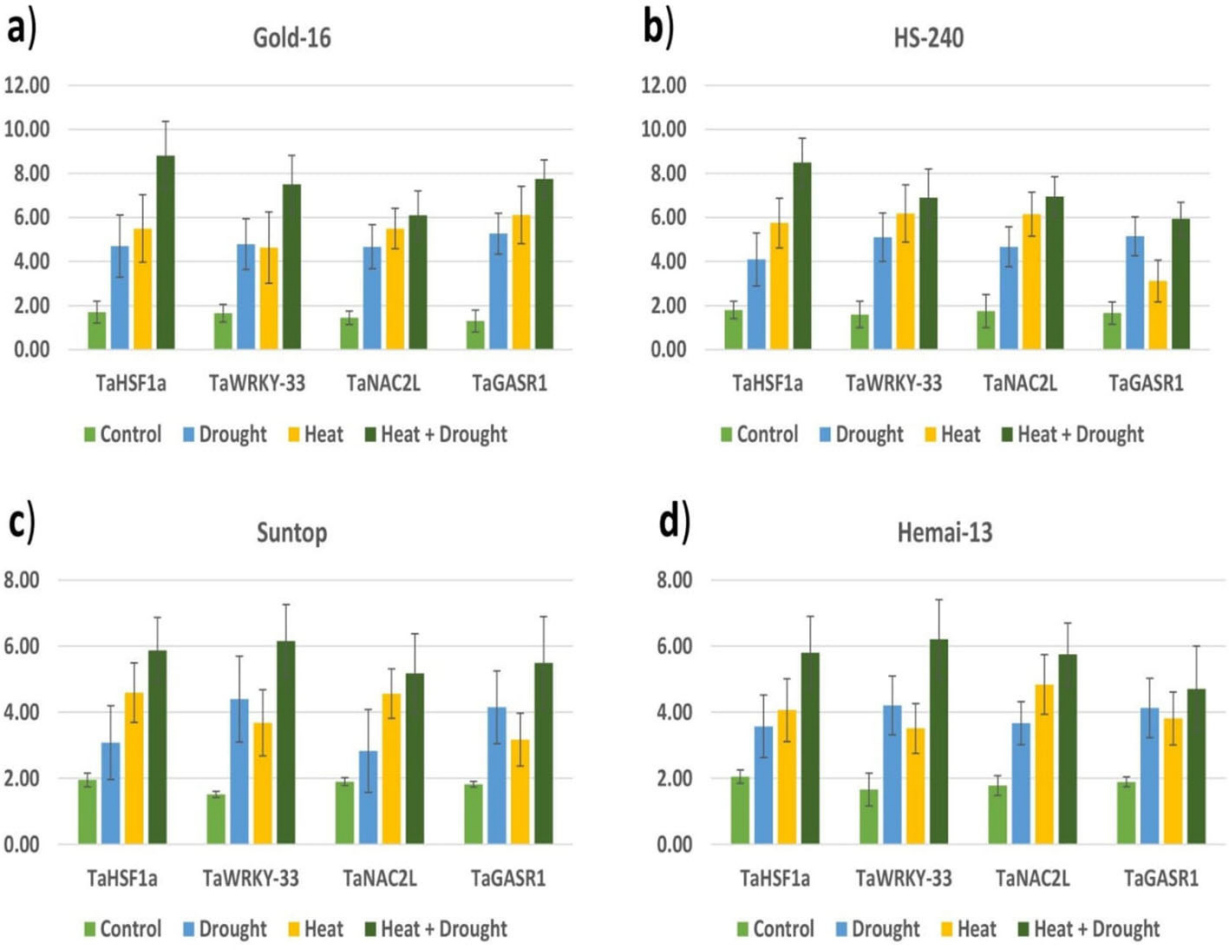
Varying expression patterns of drought- (*TaWRKY-33* and *TaGASR1*) and heat- (*TaHSF1a* and *TaNAC2L*) associated genes under the control treatment and individual and combined applications of drought and heat stress in four different wheat genotypes **(A–D)**.

All heat- and drought-related genes (*TaHSF1a*, *TaWRKY-33*, *TaNAC2L*, and *TaGASR1*) showed significant variation in their expression in all wheat genotypes under both individual and combined regimes of drought and heat stress compared with the control treatment (**Figure 9**). All genes showed upregulation in all genotypes under the stress treatments when compared with the control treatment, with significantly high relative expression in genotype Gold-16, followed by HS-240, then Suntop, and then Hemai-13 (**Figure 9**). Notably, the expression of all genes in all cultivars was more dramatic under combined drought and heat stress than under individual stresses (**Figure 9**).

## 4 Discussion

The current study evaluated the effects of individual and combined applications of drought and heat stress on the biochemical contents, physiological attributes, agronomical traits, and genetic traits of four drought-tolerant and thermostable wheat genotypes collected from different countries. Overall, all traits exhibited considerable variation under different applications of stresses. Among the biochemical traits, antioxidant enzymes (i.e., SOD, CAT, and POD) and osmolytes (i.e., TSP, TSS, and proline) demonstrated statistically distinct variations under combined and individual applications of drought and heat stress. Abiotic stresses trigger the production of ROS owing to the inhibition of the activity of antioxidant enzymes, including SOD, CAT, and POD (Shah et al., 2017). Furthermore, heat and drought stress result in the disruption of the structural integrity of protein content, the extent of which is further intensified when drought and heat stress act together (Zulkiffal et al., 2021). In addition, stress injury not only damages protein-synthesizing machinery, but also accelerates protein hydrolysis owing to increased protease activity (Vaseva et al., 2012). Similarly, under drought and heat stress the activity of enzymes is inhibited; for example, RuBisCo suppresses protein synthesis and accelerates their degradation, as described by Yang et al. (2021). In line with these observations, the current study found a consistent decrease in the activity of antioxidant enzymes (i.e., SOD, CAT, POD) and TSPs under both individual and combined applications of drought and heat stress (**Figure 1**). Conversely, Ahmadi et al. (2020) and Pour-Aboughadareh et al. (2020) recorded a consistent increase in the activity of antioxidant enzymes under salinity and drought stresses, respectively, which was probably due to the plants’ ROS scavenging ability. Moreover, plants affected by drought and heat stress accumulate more proline and compatible soluble sugars, as confirmed by various studies (Loutfy et al., 2012; Rivero et al., 2014; Hussain et al., 2018). Sattar et al. (2020) observed the increased accumulation of TSSs and proline in wheat under the combined application of drought and heat stress compared with their individual application. Correspondingly, the current study recorded a dramatic increase in TSSs and proline content under combined stresses compared with individual stresses (**Figure 1**). This can probably be attributed to the tendency of the plants to sustain redox ionic homeostasis, as proline and sugar content help to stabilize the cell membrane and various plant structures by scavenging free radicals, as described by Shah et al. (2017).

Among the physiological attributes, chlorophyll pigment content showed a reduction owing to exposure to drought and heat stress; however, this reduction was more dramatic under combined stresses (**Figure 2**). The decrease in chlorophyll content under drought and heat stress was due to the disruption of enzymes involved in chlorophyll synthesis and the increased activity of chlorophyll-degrading enzymes. This phenomenon was also observed by Shah et al. (2022) and Alghabari et al. (2021) during their studies conducted on wheat genotypes under drought and heat stress, respectively. In addition, heat stress reduces chlorophyll content by damaging the thylakoid membrane, as reported by Ristic et al. (2007). Therefore, the low level of accumulation of chlorophyll content under combined drought and heat stress was due to increased enzymatic activity or the degradation of membranes. Furthermore, the disruption of physiological machinery may alter various linked plant physiological processes such as photosynthesis (Pn), stomatal conductance (Gs), and transpiration (Tr) (Sattar et al., 2020), as reported in current study (**Figure 2**). On the other hand, the generation of ROS under abiotic stress disrupts the structural integrity of the cell membrane, leading to increased electrolyte leakage and decreased cell membrane stability (Suzuki et al., 2012). The redox stress becomes more intense when the plant is imposed to abiotic stresses, such as drought and heat, simultaneously. Therefore, in line with the findings of Shah et al. (2022) and Alghabari et al. (2021), the current study recorded a significant decrease in CMS under drought and heat stress, and this decrease was greater when these stresses were combined.

Plant–water relations and RWC are considered as better indicators of heat and drought stress than other physiological and biochemical features of plants (Farooq et al., 2019). Plant RWC indicates the water status of a plant in relation to abiotic stresses, such as heat and drought, and is highly associated with other plant relations such as water potential (ψ_w_), osmotic potential (ψ_s_), and pressure potential (ψ_p_), as reported by Sattar et al. (2020). Heat and drought stress substantially reduce the RWC owing to increased rates of transpiration, which ultimately results in the loss of water and turgor, increased solute concentration, and, subsequently, high osmotic potential (ψ_s_) (Hussain et al., 2018; Sattar et al., 2020). The current study validated these findings by recording a dramatic reduction in leaf RWC, water potential (ψ_w_), and turgor potential (ψ_p_), in addition to an increase in osmotic potential (ψ_s_), as shown in **Figure 3**. This is probably attributable to the tendency of the plants to accumulate various osmoprotectants to counter the deleterious effects of stress, as described by Rivero et al. (2014).

Reduction in flag leaf area (FLA) is an important stress-adaptive feature of plants that minimizes water loss; however, it leads to reduced Pn, Gs, and Tr (Shah et al., 2022). As a consequence, growth- and yield-related traits such as PH, NTP, SL, GPS, and TGW suffer a significant reduction (Sattar et al., 2020; Alghabari et al., 2021; Shah et al., 2022). In line with these findings, the current study recorded a statistically distinct reduction in all yield- and growth-related traits under both the combined and individual application of drought and heat stress (**Figure 4**). In addition, ultimate plant yield is a consequence of the strong correlation between biochemical and physiological events (Alsamadany, 2022; Shah et al., 2022). The decrease in TSP content under heat and drought stress leads to the loss of various proteins, which in turn triggers the chlorophyll synthesis process; hence chlorophyll degradation significantly reduces Pn and impairs the translocation of food from source to sink. Furthermore, a reduction in FLA and RWC and an increase in cell membrane damage owing to decreased SOD, POD, and CAT activity causes a significant decrease in Pn, Gs, Tr, ψ_w_, and ψ_p_ under drought and heat stress, as proved by the current study (**Figure 5**). Alsamadany (2022) found in his study, conducted on mung beans, that biochemical and physiological traits are significantly correlated in determining the crop agronomic productivity; however, the extent of the correlation varies according to type of stress and genotype. In the same way, the current study proved a significant correlation between biochemical and physiological traits in determining the ultimate agronomic productivity of wheat genotypes; however, the degree of correlation and expression of these traits varied depending on the type of stress and genotype, as proved by the PCA (**Figure 6**) and heatmap analysis (**Figure 7**). Furthermore, mineral distribution is disrupted in plants facing drought and heat stress, which in turn disrupts the enzymatic activities involved in starch synthesis within the leaves, as reviewed by Fahad et al. (2017). Correspondingly, the SEM micrograph shows a clear difference in mineral distribution and starch granulation between plants exposed to heat and drought stress and those in the control group (**Figure 8**). Interestingly, all genotypes depicted variable response in terms of trait association and expression under individual and combined applications of stress. This was probably due to the different genetic structures of genotypes under study. As a result, an expression analysis of drought- and heat-associated genes was conducted. All genes (i.e., *TaHSF1a*, *TaWRKY-33*, *TaNAC2L*, and *TaGASR1*) were over-expressed in the plants under the individual and combined application of drought and heat stress; however, the expression patterns revealed significantly higher levels of gene expression under combined treatment in all wheat genotypes (**Figure 9**). The high level of gene expression in a genotype is an indicator of its robust tolerance mechanism to stress (Shah et al., 2017). Shah et al. (2022) similarly observed the high level of expression of *TaGASR1* and *TaWRKY-33* when plants were grown under drought and combined treatments, compared with heat and control treatments. The *TaGASR1* and *WRKY* genes tend to activate the redox ionic homeostasis in plants by triggering various antioxidant mechanisms involved in ABA-mediated signaling (Niu et al., 2012; Zhang et al., 2017). As combined drought and heat intensify the oxidative stress, the plant responds with high levels of expression of certain genes, as recorded in the present study. On the other hand, *TaNAC2L* and *TaHSF1a* showed comparatively high levels of expression in plants grown under treatments of heat and combined stress compared with drought stress and the control treatment (**Figure 9**). These findings were consistent with the findings of Guo et al. (2015) and Shah et al. (2022). Plants counter the effect of stress by increasing the production of various osmoprotectants, including heat shock proteins, and regulating the expression of various genes involved in counter-stress pathways (He et al., 2018). Drought and heat stress collectively create enough stress to trigger the production of more osmoprotectants and translational and post-translation modulations (Zeng et al., 2022). This is the most probable reason for the high relative level of gene expression in the plants grown under the combined application of drought and heat stress compared with individual treatments. Overall, the relative level of gene expression remained the highest in the genotype Gold-16 followed by HS-20, then Suntop, and then Hemai-13 under both the individual and combined application of stresses. The current study concludes that plants are not passive entities; they react to environmental stresses by modulating biochemical, physiological, morphological, and molecular processes. The tendency of plants to retain morphophysiological and agronomical integrity is potentially enough to mitigate the hazardous impacts of drought and heat stresses, as proved by Gold-16 in current study. In addition, the high levels of expression of genes *TaHSF1a, TaWRKY-33, TaNAC2L*, and *TaGASR1* is associated with the tendency to respond quickly to abiotic stresses through modulating the levels of different osmoprotectants and antioxidant enzymes. This helps the plant to sustain a physiological and biochemical equilibrium and produce a high agronomic yield. Therefore, the current study recommends further thorough evaluation of the Gold-16 genotype *via* large trials in arid and semiarid regions of the world where drought and heat stresses are potential problems at the anthesis and grain-filling stages of wheat production.

## Data availability statement

The original contributions presented in the study are included in the article/supplementary material. Further inquiries can be directed to the corresponding authors.

## Author contributions

HA and YA conceived and executed the experiment. ZS performed the analysis and wrote the manuscript. All authors contributed to the article and approved the submitted version.
